# Adaptive Heterogeneity-Aware Tensor Decomposition for Hyperspectral Image Denoising

**DOI:** 10.3390/s26144516

**Published:** 2026-07-16

**Authors:** Jiaxian Long, Chaowei Yuan

**Affiliations:** School of Information and Communication Engineering, Beijing University of Posts and Telecommunications, Beijing 100876, China

**Keywords:** hyperspectral image denoising, tensor decomposition, region-aware processing, spectral–spatial modeling, adaptive weighted shrinkage, remote sensing, geospatial analysis

## Abstract

Hyperspectral image denoising must reconcile global spectral coherence with spatially heterogeneous scene content. This paper presents Adaptive Heterogeneity-Aware Tensor Decomposition (AHTD), a refinement module that augments a global Tucker initialization with heterogeneity-guided local tensor shrinkage. The method estimates a global spectral subspace, detects heterogeneous regions via combined variance and edge analysis, and applies adaptive weighted singular-value shrinkage modulated by regional patch complexity. To isolate the effect of the heterogeneity-aware selection mechanism itself, experiments employ a controlled internal ablation protocol comparing three pipeline variants under identical noise realizations, ranks, and parameter settings on the Pavia_80, Indian Pines corrected, and Salinas corrected benchmarks under synthetic mixed noise (σ=0.03 Gaussian with stripe and impulse noise): a global Tucker baseline, a dense full-local refinement variant, and the proposed selective AHTD. AHTD achieves peak signal-to-noise ratio (PSNR) gains of 0.35–0.40 dB over the global Tucker baseline while maintaining or improving structural similarity index (SSIM), spectral angle mapper (SAM), and relative dimensionless global error in synthesis (ERGAS), at approximately threefold lower runtime than dense full-local refinement, demonstrating its value as a computationally efficient, interpretable refinement stage for tensor-based hyperspectral processing. We note that matched comparisons against externally published denoising methods are not included in this study; these results establish the benefit of heterogeneity-aware selective refinement within the proposed Tucker-based pipeline.

## 1. Introduction

Hyperspectral imaging captures contiguous spectral measurements for every pixel, enabling accurate material identification and scene interpretation. This rich data supports applications such as agricultural monitoring, mineral exploration, environmental assessment, urban mapping, and disaster response. However, hyperspectral imagery is frequently degraded by mixed noise from sensors, atmosphere, and calibration errors. Typical corruptions include Gaussian noise, striping and dead-line artifacts, impulse disturbances, and band-dependent spectral distortions. These complex degradations challenge denoising methods, which must preserve spatial detail and spectral fidelity because restored data underpin downstream tasks such as land-cover classification and target detection.

Hyperspectral bands are strongly correlated due to material signatures and sensor sampling, but effective denoising must also handle device-specific artifacts (e.g., pushbroom striping, detector non-uniformity) and acquisition-dependent distortions. Early subspace-based and bandwise collaborative filters exploit spectral redundancy, yet matrix low-rank recovery methods destroy the native spatial–spectral structure by unfolding the cube into a matrix. Recent tensor methods and spatial-regularization approaches demonstrate the benefits of modeling spectral–spatial structure jointly, preserving the full three-way data geometry.

Tensor decomposition approaches naturally model joint spatial–spectral correlations and have become a mainstream direction. Region-aware tensor methods further extend this strategy by adapting processing to local scene heterogeneity, refining complex regions while applying simpler models to homogeneous areas. However, prior region-aware methods still face key limitations:

1. Region identification commonly relies on fixed thresholds or heuristics, making it sensitive to noise level and scene composition.
2. Local tensor ranks are often predetermined or uniform, which cannot adapt to local complexity differences between textured and smooth regions.
3. Existing region-aware pipelines often alternate between global and local updates without a formal convergence guarantee, undermining theoretical assurances of stable behavior.

We propose an Adaptive Heterogeneity-Aware Tensor Decomposition (AHTD) framework that combines a global Tucker initialization with adaptive local refinement informed by heterogeneity cues.

Our contributions are:

- A heterogeneity-guided region detection that combines local variance and spatial edge responses to identify complex spatial regions.
- An adaptive patch-wise shrinkage mechanism that adjusts singular-value suppression per patch based on heterogeneity.
- A selective hybrid pipeline that applies local refinement only on high-complexity regions to reduce computation.
- A heterogeneity-weighted fusion scheme that blends global and local estimates while preserving spectral consistency.
- A controlled experimental evaluation on three standard benchmarks with standardized metrics—peak signal-to-noise ratio (PSNR), structural similarity index (SSIM), spectral angle mapper (SAM), and relative dimensionless global error in synthesis (ERGAS)—using a protocol-matched internal ablation design to isolate the effect of the heterogeneity-aware selection mechanism.

Scope and limitations of this study: The experiments in this paper employ a controlled internal ablation protocol: three variants of the same tensor-based pipeline (a global Tucker baseline, a dense full-local refinement variant, and the proposed selective AHTD) are compared under identical noise realizations, Tucker ranks, patch settings, and evaluation code. This design isolates the effect of the heterogeneity-aware selection mechanism itself, so that any observed difference can be attributed to that mechanism rather than to differences in implementation, parameter tuning, or preprocessing. Matched numerical comparisons against externally published denoising methods (such as block-matching and 3D filtering (BM3D)/block-matching and 4D filtering (BM4D), Low-Rank Matrix Recovery (LRMR), nonlocal low-rank tensor methods, tensor-ring decomposition, total variation (TV)-regularized methods, or deep-learning approaches) are not included in this study; such comparisons require fully matched reproduction under identical protocols and represent an important direction for future work. The results reported here demonstrate that heterogeneity-aware selective refinement provides consistent improvements over the global Tucker baseline and offers a substantial runtime advantage over dense full-local refinement within the proposed pipeline.

The remainder of the paper is organized as follows. Section 2 reviews related work on hyperspectral image (HSI) denoising. Section 3 describes the AHTD framework. Section 4 presents the experimental results. Section 5 reports ablation analyses. Section 6 concludes the paper.

## 2. Related Work

### 2.1. Literature Context

HSI denoising has been addressed with both model-based and data-driven approaches, which offer complementary strengths.

Spectral subspace and filtering methods: Spectral-subspace methods exploit low-rank structure in the spectral domain, where adjacent bands are highly correlated. These methods estimate a low-dimensional spectral basis and restore spatial content in that reduced space. Bandwise collaborative filters such as block-matching and 3D filtering (BM3D) [1] and its volumetric extension block-matching and 4D filtering (BM4D) [2] exploit spatial self-similarity within single bands or spatiospectral volumes, though applying these filters independently per band can weaken spectral coherence.

Matrix and tensor low-rank models: Matrix-based recovery algorithms such as Low-Rank Matrix Recovery (LRMR) [3] represent the hyperspectral cube as a low-rank matrix plus sparse corruption. While effective, matrix unfolding destroys the native 3D structure and can impair spatial–spectral consistency. Tensor decomposition methods retain the full three-way structure and are widely used in HSI denoising [4,5,6,7]. A nonlocal tensor-ring decomposition approach for HSI denoising has also been proposed [8], demonstrating competitive restoration on real-world remote sensing data. Global and nonlocal low-rank factorizations have been combined to exploit both spectral low-rankness and spatial self-similarity [9]. Extensions such as spectral–spatial sparse representation [10], spatial–spectral TV-regularized tensor decomposition [11], total variation regularized matrix factorization [12], and TV-regularized low-rank tensor methods [6] improve robustness to complex noise. Spectral–spatial prior fusion [13] and nonlocal prior-based denoising [14] also highlight the value of combining complementary regularization strategies. Multiscale low-rank tensor decomposition has been studied for hyperspectral dimensionality reduction [15], offering insights for structured tensor modeling. Still, many tensor methods impose uniform low-rank constraints across the entire image, which limits their ability to adapt to spatial heterogeneity.

Region-aware and localized methods: Region-aware processing adapts to local scene complexity. Representative approaches include nonlocal low-rank tensor decomposition [4], patch-based sparse representation methods [10], and spatially regularized low-rank models [12]. Hybrid strategies that combine global initialization with localized patch refinement in heterogeneous regions have also been explored. This hybrid approach reduces over-smoothing in complex areas but typically relies on fixed thresholding and preset shrinkage settings. Our method differs by introducing a heterogeneity mask built from local variance and edge responses, and by using adaptive patch-wise shrinkage governed by patch heterogeneity. While recent adaptive tensor models support data-driven refinement, a complete region-aware framework that unifies adaptive region detection and practical optimization formulation remains underdeveloped.

Deep learning approaches: Deep networks such as HCANet [16] learn spectral–spatial priors from training data and often achieve competitive performance on noisy scenes. Recent studies include HDST [17], RND [18], ILRNet [19], and adaptive fidelity/prior balancing [20]. We cite these works for context only and do not directly compare AHTD to their reported numbers, since their training corpora and evaluation protocols differ from our internal protocol, and matched reproduction of these methods is outside the scope of the present study.

### 2.2. Evaluation Design

Because published denoising results are sensitive to noise-model details, preprocessing, and parameter tuning, direct numerical comparison to externally reported numbers can be misleading without a fully matched reproduction. The experiments in this paper therefore adopt a protocol-matched internal ablation design: AHTD is evaluated against a global Tucker baseline and a dense full-local-refinement variant under identical noise realizations, ranks, and patch settings, so that any observed difference is attributable to the proposed heterogeneity-aware selection mechanism itself. This controlled design is appropriate for isolating the contribution of the proposed selection mechanism. Representative external methods are cited in the literature review above to contextualize our work; matched numerical comparisons against them are outside the scope of the present study.

Our method builds on region-aware tensor decomposition ideas. We integrate adaptive region detection, localized patch-wise shrinkage refinement, and soft fusion to achieve selective enhancement in heterogeneous regions.

## 3. Proposed Method

### 3.1. Global Correlation and Local Heterogeneity

Hyperspectral images exhibit strong cross-band correlation and usually admit a compact low-dimensional representation. This global property is useful for coarse noise suppression. In contrast, the spatial domain is often heterogeneous, with textures, boundaries, and mixed land-cover types that violate the stationarity assumptions of uniform global models.

Our method separates these two regimes by estimating a global tensor initialization first and then applying localized tensor decomposition only to spatial regions where the global model shows higher heterogeneity.

### 3.2. Notation and Problem Formulation

Notation: We follow standard tensor notation [7]. A third-order tensor is written as a calligraphic letter, e.g., $Y\in R^{M\times N\times B}$, where *M* and *N* are spatial dimensions (rows and columns) and *B* is the number of spectral bands. The mode-*n* unfolding (matricization) of Y is denoted by $Y_{\left(n\right)}\in R^{I_{n}\times \prod_{j\neq n}I_{j}}$, which rearranges the tensor fibers into a matrix. The mode-*n* tensor–matrix product is written as $\times_{n}$. The Frobenius norm is ${∥Y∥}_{F}=\sqrt{\sum_{i,j,b}Y{\left(i,j,b\right)}^{2}}$, and the nuclear norm of a matrix A is ${∥A∥}_{*}$, defined as the sum of its singular values. Bold uppercase letters denote matrices and 2D spatial maps (e.g., U, H), bold lowercase letters denote vectors (e.g., u), italic letters denote scalars (e.g., α, *C*), and subscripts in parentheses denote mode indices.

Let $Y\in R^{M\times N\times B}$ denote the noisy hyperspectral tensor. The clean image is X and the additive noise is N:

(1) Y=X+N.

### 3.3. Global Tensor Initialization

We begin by estimating a global tensor approximation of the noisy hyperspectral cube. This stage uses Tucker decomposition to capture the dominant spectral–spatial structure of the full tensor without flattening it into a single matrix. The global approximation provides a structured baseline that preserves the principal components of the data across all three modes.

Specifically, we compute a global Higher-Order Singular Value Decomposition (HOSVD)-based Tucker decomposition on the full tensor:

(2) $$Y\approx G\times_{1}U_{1}\times_{2}U_{2}\times_{3}U_{3},$$

where $U_{1}$ and $U_{2}$ are factor matrices for the two spatial modes (rows and columns, respectively), and $U_{3}$ is the factor matrix for the spectral mode. The corresponding core tensor G captures the interaction among the selected multilinear ranks.

The resulting global estimate suppresses noise while retaining the dominant tensor structure of the hyperspectral cube. We denote the reconstructed Tucker approximation by

(3) $$Y_{\mathrm{pre}}=G\times_{1}U_{1}\times_{2}U_{2}\times_{3}U_{3}.$$

This reconstructed approximation preserves the native 3D representation and avoids flattening the data into a matrix, keeping subsequent processing aligned with tensor operations. The heterogeneity score is computed from the global estimate; a small 3×3 median filter is applied to a copy of the global estimate before scoring to stabilize region selection against residual noise spikes, without modifying $Y_{\mathrm{pre}}$ itself.

Compared with deep learning approaches, AHTD requires no task-specific training data, offers an interpretable tensor-decomposition structure, and incurs moderate, deterministic computational overhead relative to a global baseline. These properties may be attractive in scenarios where training samples are scarce or physical interpretability is valued, though we do not experimentally compare against deep-learning methods in this study.

### 3.4. Heterogeneity-Based Region Identification

The global baseline estimate reveals regions where the global tensor initialization is less confident. These regions are identified by a heterogeneity score computed from the global baseline itself, rather than from a residual tensor.

We compute a bandwise variance map and a spatial edge map from the median-filtered copy of the global estimate (denoted ${\tilde{Y}}_{\mathrm{pre}}$), which stabilizes scoring against residual noise spikes:

(4) $$\begin{array}{cc} V(i,j) & =\frac{1}{B-1}\sum_{b=1}^{B}{\left({\tilde{Y}}_{\mathrm{pre}}\left(i,j,b\right)-{\bar{\tilde{Y}}}_{\mathrm{pre}}\left(i,j\right)\right)}^{2}, \end{array}$$

(5) $$\begin{array}{cc} E(i,j) & =\frac{1}{B}\sum_{b=1}^{B}\sqrt{{\left(\partial_{x}{\tilde{Y}}_{\mathrm{pre}}\left(i,j,b\right)\right)}^{2}+{\left(\partial_{y}{\tilde{Y}}_{\mathrm{pre}}\left(i,j,b\right)\right)}^{2}}, \end{array}$$

where ${\bar{\tilde{Y}}}_{\mathrm{pre}}\left(i,j\right)$ is the bandwise mean of the filtered copy and $\left(\partial_{x},\partial_{y}\right)$ are spatial gradients computed per band. Both maps are independently normalized to [0,1] via min–max scaling (denoted norm(·)) and combined into a heterogeneity score:

(6) H(i,j)=α·norm(V(i,j))+β·norm(E(i,j)),

with weights α and β controlling the relative emphasis on local variance and edge strength. In our implementation, we set α=0.6 and β=0.4, which was found to provide a balanced combination across the three benchmark datasets: the variance map captures smooth intensity transitions while the edge map highlights sharp edges. These values were determined empirically and held fixed throughout all experiments; a more exhaustive sensitivity analysis of these weights is deferred to future work.

The heterogeneity mask is obtained by thresholding the normalized score:

(7) $$M\left(i,j\right)=\left\{\begin{array}{cc} 1, & H\left(i,j\right)>T_{H},\\ 0, & \mathrm{otherwise}, \end{array}\right.$$

where $T_{H}$ is chosen adaptively as the 70th percentile of the heterogeneity score distribution H, which by construction selects pixels whose heterogeneity score falls in the top 30% for local refinement. Using a percentile-based threshold (rather than a fixed absolute value) allows the mask to adapt to the score distribution of each image without per-dataset tuning: the resulting approximately 30% coverage provides a balanced trade-off between refinement coverage and computational cost, while visual inspection confirms that the selected pixels consistently correspond to edges, boundaries, and textured regions across datasets and noise levels. This mask therefore focuses refinement on complex structures while leaving homogeneous areas to the global baseline.

### 3.5. Localized Patch-Wise Refinement

For each patch centered on a masked pixel, we extract a local tensor $P_{k}\in R^{P\times P\times B}$ from the noisy image Y, where *P* denotes the spatial patch size (P=25 in our implementation), and refine it with adaptive weighted shrinkage on each mode unfolding. We note that heterogeneous regions in hyperspectral images often have irregular shapes; the use of square patches is a practical approximation that simplifies tensor decomposition and patch aggregation. Overlapping patches (stride of 5 pixels in our implementation) are used to reduce boundary artifacts at patch edges. Refined patches are accumulated into a local estimate $Y_{\mathrm{local}}$ via overlapping patch aggregation, where each pixel’s value is averaged across all patches covering it (using a count map $N_{\mathrm{acc}}$ for normalization); the soft fusion scheme (Section 3.8) then blends the local estimate with the global baseline to ensure smooth transitions between refined and unrefined regions.

(8) $$P_{k}=L_{k}+N_{k},$$

where $L_{k}$ is the clean local tensor and $N_{k}$ is the local noise term.

Noise variance estimation: Before describing the shrinkage operator, we first specify how the noise variance is estimated. The noise standard deviation $\hat{\sigma }$ is estimated via the median absolute deviation (MAD) estimator applied directly to the global baseline residual $R=Y-Y_{\mathrm{pre}}$: $\hat{\sigma }=\mathrm{median}(|R-\mathrm{median}\left(R\right)|)\;/\;0.6745$, where the factor 0.6745 calibrates the MAD to a standard-deviation estimate under Gaussian noise. The MAD estimator is inherently robust to outliers (including edge pixels and heterogeneous structures), so it does not require a priori heterogeneity identification. Because the singular values of a mode-*n* unfolding scale with the square root of the number of columns in that unfolding, the pixel-domain variance ${\hat{\sigma }}^{2}$ is scaled by the number of columns of the mode-*n* unfolding matrix (denoted $J_{n}=\prod_{j\neq n}I_{j}$ for a patch of dimensions $I_{1}\times I_{2}\times I_{3}$) before being used in the shrinkage operator below, yielding the mode-adapted noise level ${\hat{\sigma }}^{2}\cdot J_{n}$.

Mathematical rule: Each mode unfolding is decomposed via singular value decomposition (SVD). Given the mode-*n* unfolding matrix $P_{k(n)}$ of local patch $P_{k}$, its full SVD is computed (no pre-truncation) as $L_{n}\Sigma_{n}R_{n}^{T}$, where $\Sigma_{n}=\mathrm{diag}\left(S_{1},S_{2},\ldots ,S_{r_{n}}\right)$ with $r_{n}$ being the total number of non-zero singular values (i.e., the matrix rank, equal to $\min(I_{n},J_{n})$ where $J_{n}=\prod_{j\neq n}I_{j}$ for a patch of size P×P×B), and $L_{n}$, $R_{n}$ are the left and right singular vector matrices, respectively. The adaptive shrinkage operator then applies a heterogeneity-aware penalty:

(9) $$S_{i}^{\mathrm{new}}=\max\left(S_{i}-\frac{C\cdot {\hat{\sigma }}^{2}\cdot J_{n}}{S_{i}+\varepsilon },0\right),$$

where ${\hat{\sigma }}^{2}\cdot J_{n}$ is the mode-adapted noise variance (scaled by the number of columns $J_{n}$ of the mode-*n* unfolding to match the SVD singular-value scale), *C* is a modulation coefficient determined by the patch’s heterogeneity weight, and ε>0 is a small constant (set to $10^{-8}$ in our implementation) to prevent division by zero for near-zero singular values.

Computation of *C*: In the implemented prototype, the patch heterogeneity is approximated from the average heterogeneity score within the patch. Let ${\bar{H}}_{k}$ denote the mean heterogeneity score inside patch *k*, normalized to [0,1]. The modulation coefficient is set as

(10) $$C=C_{\max}-\left(C_{\max}-C_{\min}\right)\cdot {\bar{H}}_{k},$$

with $C_{\max}=1.0$ and $C_{\min}=0.2$ being preset bounds (chosen so that high-heterogeneity patches retain most singular components while low-heterogeneity patches apply stronger shrinkage). Consequently, higher-heterogeneity patches (large ${\bar{H}}_{k}$) receive smaller *C* and preserve more singular components, whereas smoother patches (small ${\bar{H}}_{k}$) receive larger *C* and are denoised more aggressively. This contrasts with fixed-rank methods that truncate singular values at a uniform cutoff regardless of local complexity.

Intuitive effect: Consider a patch straddling a road–vegetation boundary: its mean heterogeneity score ${\bar{H}}_{k}$ is high, *C* is small, and the dominant singular components associated with the edge are retained. In a homogeneous agricultural field patch, the mean heterogeneity score is low, *C* is large, and the low-energy singular components dominated by noise are suppressed more strongly. The reconstructed patch ${\hat{P}}_{k}$ is then obtained by averaging the three shrunken mode-wise reconstructions.

### 3.6. Adaptive Shrinkage Behavior

Selecting the local shrinkage strength is critical for balancing denoising strength with structural fidelity. In AHTD, the local penalty is initialized from the global tensor estimate and modulated by patch heterogeneity. Specifically, patches with higher heterogeneity receive smaller penalties in the shrinkage rule, allowing more singular values to be retained and thus preserving fine textures and edges. More homogeneous patches use stronger shrinkage to suppress residual noise, since uniform regions benefit from aggressive low-rank approximation. This adaptive modulation contrasts with standard Tucker decomposition, which applies uniform rank truncation across the entire image regardless of local complexity. The modulation coefficient *C* (Equation (10)) translates the heterogeneity score into a continuous shrinkage scaling factor, ensuring that the transition between strong and weak denoising is smooth rather than abrupt.

### 3.7. Computational Complexity and Implementation Notes

The computational cost of AHTD consists of three main components: global tensor initialization, local patch refinement, and weighted fusion.

Global Tucker initialization: The HOSVD-based Tucker decomposition requires computing the SVD of each mode unfolding. For mode-*n* unfolding of size $I_{n}\times \prod_{j\neq n}I_{j}$, the reduced SVD cost is $O(I_{n}^{2}\prod_{j\neq n}I_{j})$ when $I_{n}<\prod_{j\neq n}I_{j}$. For the spatial modes (n=1,2), this yields $O(M^{2}NB)$ and $O(N^{2}MB)$, respectively; for the spectral mode (n=3), the cost is $O(MNB^{2})$. The core tensor computation adds $O(MNB\bar{r})$ with multilinear ranks $\left(r_{1},r_{2},r_{3}\right)$ satisfying $\bar{r}=\max\left(r_{1},r_{2},r_{3}\right)\ll \min\left(M,N,B\right)$. The dominant cost in typical hyperspectral settings scales with the product of spatial and spectral dimensions.

Local patch refinement: For each masked patch of size P×P×B, the per-patch cost is dominated by the SVD of three mode unfoldings. The spatial unfoldings (mode-1 and mode-2) have dimensions P×PB, with SVD cost $O(P^{3}B)$ each; the spectral unfolding (mode-3) has dimension $B\times P^{2}$, with SVD cost $O(P^{2}B^{2})$ when $B<P^{2}$. The dominant per-patch cost is therefore $O(P^{2}B^{2}+P^{3}B)$. With $N_{p}$ masked patches, the total local cost is $O(N_{p}\left(P^{2}B^{2}+P^{3}B\right))$. The patches are extracted with stride 5 (overlapping), and the adaptive mask selects approximately 30% of patch centers, yielding $N_{p}\approx 0.3\cdot \left(M/5\right)\left(N/5\right)=0.3\;MN/25$ in our experiments. Because only a subset of patch centers is processed locally, the total runtime is substantially lower than that of a full dense refinement over the entire cube.

Fusion: The soft fusion is an element-wise weighted average, costing O(MNB), which is negligible relative to the other components.

In practice, the adaptive mask typically covers roughly one third of the image area on standard datasets, which keeps refinement selective while still affecting the most heterogeneous structures.

The current AHTD prototype is implemented in Python (v3.10) using NumPy (v1.24) and SciPy (v1.10), with core tensor operations vectorized and implemented through matrix factorization routines. The 3×3 median filter mentioned above is applied only to a copy of the global baseline estimate for heterogeneity scoring and does not directly modify the tensors used in the fusion step.

### 3.8. Adaptive Fusion

The global estimate and the local refinement are fused with a soft heterogeneity weight derived from the normalized heterogeneity score. Let $Y_{\mathrm{local}}$ denote the refined patch aggregation and let w(i,j)=H(i,j) denote the normalized heterogeneity score (which lies in [0,1] by construction, since norm(V),norm(E)∈[0,1] and α+β=1); the final estimate is then

(11) $$Y_{\mathrm{final}}\left(i,j,b\right)=\left(1-w\left(i,j\right)\right)\cdot Y_{\mathrm{pre}}\left(i,j,b\right)+w\left(i,j\right)\cdot Y_{\mathrm{local}}\left(i,j,b\right),$$

where the weight w(i,j) is applied uniformly across all spectral bands *b*. This soft fusion preserves the global structure in homogeneous areas while allowing local refinement to dominate where the heterogeneity score is high.

### 3.9. Optimization Objective and Practical Behavior

We formulate the denoising objective as a conceptual surrogate that motivates the implemented refinement stage:

(12) $$F\left(X\right)=\frac{1}{2}{∥Y-X∥}_{F}^{2}+\mu \sum_{k}\left(1-{\bar{H}}_{k}\right)\sum_{n=1}^{3}{∥X_{k(n)}∥}_{*},$$

where $X_{k(n)}$ denotes the mode-*n* matricization of the local tensor restricted to patch *k*, the inner sum runs over all three modes (n=1,2,3), ${∥\cdot ∥}_{*}$ is the nuclear norm (sum of singular values) promoting low-rank structure within each patch, μ>0 is a penalty weight controlling the overall strength of low-rank regularization, and ${\bar{H}}_{k}\in \left[0,1\right]$ is the mean heterogeneity score within patch *k*. The first term enforces data fidelity. The second term encourages local low-rank structure with patch-dependent strength: the factor $\left(1-{\bar{H}}_{k}\right)$ reduces the nuclear-norm penalty in high-heterogeneity patches (allowing more singular components to be retained for textures and edges) and increases it in homogeneous patches (applying stronger shrinkage). In the implemented algorithm, this heterogeneity-dependent modulation is realized through the adaptive shrinkage coefficient *C* (Equation (10)), which continuously scales the singular-value threshold as a function of patch heterogeneity, rather than through explicit iterative minimization of the objective above.

Note on optimization: This objective is not directly minimized by Algorithm 1. The implemented AHTD prototype uses a single-pass selective refinement as a practical approximation to the heterogeneity-weighted objective above. In the current prototype, local refinement is executed as a single selective pass over masked patches: each selected patch is processed with adaptive weighted shrinkage and then blended back into the full estimate. This design avoids repeated global iterations, keeps runtime predictable, and matches the behavior of the implemented prototype.

Specifically, the adaptive weighted shrinkage applied to each masked patch in Algorithm 1 implements a singular-value thresholding operation whose threshold is modulated by the heterogeneity coefficient *C*, and the final soft fusion approximates the balance between the fidelity and heterogeneity-weighted terms. We include this formulation to clarify the conceptual motivation behind the design choices (e.g., why shrinkage strength depends on heterogeneity), rather than as a formal objective that is iteratively optimized.

The single-pass design guarantees that the refinement stage terminates in bounded time and does not diverge from the stable global initialization. To empirically verify the stability of the single-pass design, we repeated the full AHTD pipeline 10 times on each benchmark dataset with identical noise realizations. The per-pixel standard deviation of the output was below $10^{-6}$ (normalized), confirming that the deterministic single-pass procedure produces highly consistent results across runs and that the overlapping patch aggregation introduces no stochastic artifacts. The lack of a formal convergence proof for iterative or alternating schemes that incorporate heterogeneity-weighted penalties is acknowledged as a limitation in Section 5.7.

### 3.10. Algorithm Summary

The complete AHTD workflow is presented in Algorithm 1.
**Algorithm 1** Adaptive Heterogeneity-Aware Tensor Decomposition (AHTD)**Require:** Noisy tensor Y, global Tucker ranks $\left(r_{1},r_{2},r_{3}\right)$, patch size *P*, patch stride *s*, heterogeneity weights α,β, heterogeneity percentile threshold (to determine $T_{H}$), shrinkage bounds $C_{\max},C_{\min}$, stability constant ε.**Ensure:** Denoised tensor $Y_{\mathrm{final}}$.  1:**Global initialization:** Compute Tucker decomposition $Y\approx G\times_{1}U_{1}\times_{2}U_{2}\times_{3}U_{3}$ with ranks $\left(r_{1},r_{2},r_{3}\right)$; reconstruct $Y_{\mathrm{pre}}$ via Equation (3); estimate pixel-domain noise variance ${\hat{\sigma }}^{2}$ from the global baseline residual using the MAD estimator (Section 3.5).  2:**Heterogeneity analysis:** Apply a 3×3 median filter to a copy of $Y_{\mathrm{pre}}$ to obtain ${\tilde{Y}}_{\mathrm{pre}}$ for scoring stability; compute variance map V and edge map E from ${\tilde{Y}}_{\mathrm{pre}}$ via Equations (4) and (5); form score H via Equation (6); derive mask M via Equation (7). The median filter is used only for heterogeneity scoring and does not modify the $Y_{\mathrm{pre}}$ used in fusion.  3:Initialize local estimate $Y_{\mathrm{local}}\leftarrow 0$ and count map $N_{\mathrm{acc}}\leftarrow 0$ (both of size M×N×B and M×N, respectively).  4:**for** each patch $P_{k}$ centered at (i,j) where M(i,j)=1, sampled with stride *s*, extracted from the noisy image Y **do**  5:    For each mode n=1,2,3, compute the SVD of the mode-*n* unfolding $P_{k(n)}$; apply adaptive singular-value shrinkage via Equation (9) with *C* from Equation (10) using the mode-adapted noise level ${\hat{\sigma }}^{2}\cdot J_{n}$; fold back to obtain the mode-*n* shrunken reconstruction. Average the three mode-wise reconstructions to obtain ${\hat{P}}_{k}$.  6:    Accumulate ${\hat{P}}_{k}$ into the local estimate at corresponding global positions: for each pixel (i,j) in patch *k* with local coordinates $\left(i^{\prime },j^{\prime }\right)$, update $Y_{\mathrm{local}}\left(i,j,b\right)+={\hat{P}}_{k}\left(i^{\prime },j^{\prime },b\right)$ and increment count map $N_{\mathrm{acc}}\left(i,j\right)$.  7:**end for**  8:Normalize by the count for pixels covered by at least one patch: $Y_{\mathrm{local}}\left(i,j,b\right)=Y_{\mathrm{local}}\left(i,j,b\right)/N_{\mathrm{acc}}\left(i,j\right)$ where $N_{\mathrm{acc}}\left(i,j\right)>0$; for uncovered pixels set $Y_{\mathrm{local}}\left(i,j,b\right)=Y_{\mathrm{pre}}\left(i,j,b\right)$ so that fusion reduces to the global baseline.  9:**Fusion:** Let w(i,j)=H(i,j) denote the normalized heterogeneity score (in [0,1] by construction); fuse via $Y_{\mathrm{final}}\left(i,j,b\right)=\left(1-w\left(i,j\right)\right)\cdot Y_{\mathrm{pre}}\left(i,j,b\right)+w\left(i,j\right)\cdot Y_{\mathrm{local}}\left(i,j,b\right)$ (Equation (11)). 10:**return** $Y_{\mathrm{final}}$.

## 4. Experimental Results and Analysis

### 4.1. Datasets and Experimental Setup

Consistent with the controlled internal ablation protocol described in Section 1, this section compares three pipeline variants (global Tucker baseline, dense full-local refinement, and proposed selective AHTD) on three standard hyperspectral datasets under identical noise realizations, ranks, and parameter settings. The purpose of this design is to isolate the effect of the heterogeneity-aware selection mechanism itself. Matched comparisons against externally published denoising methods are not included (see the Scope and limitations paragraph in Section 1).

The datasets are:

- Pavia_80 (200×200×80)—a smaller urban hyperspectral cube with mixed scene structure;
- Indian Pines corrected (145×145×200)—agricultural fields with diverse crop and soil textures;
- Salinas corrected (512×217×204)—a high-resolution agricultural scene with dense spatial detail.

In addition to experimental evaluations, we perform qualitative validation on raw, uncorrected hyperspectral cubes (Indian_pines.mat, Pavia.mat, Salinas.mat) to demonstrate the pipeline’s behavior on real sensor data when clean ground truth is unavailable.

For experimental evaluation, we corrupt clean data with synthetic mixed noise comprising additive zero-mean Gaussian noise (σ=0.03), random stripe noise affecting 4% of columns, and impulse noise affecting 2% of pixels. All images are normalized to [0,1]. To additionally assess performance under higher noise levels, we test two elevated Gaussian levels, σ=0.05 and σ=0.10, while keeping the same stripe and impulse settings. This synthetic noise model approximates common sensor and transmission artifacts in remote sensing scenes.

We use four standard metrics for quantitative evaluation:

- Peak signal-to-noise ratio (PSNR);
- Structural similarity index (SSIM);
- Spectral angle mapper (SAM);
- Relative dimensionless global error in synthesis (ERGAS).

These metrics quantify both spatial restoration and spectral fidelity.

Following established evaluation practices, we report results on multiple datasets and provide descriptions of the evaluation protocol for reproducibility.

### 4.2. Qualitative Visual Comparison

**Figure 1:**
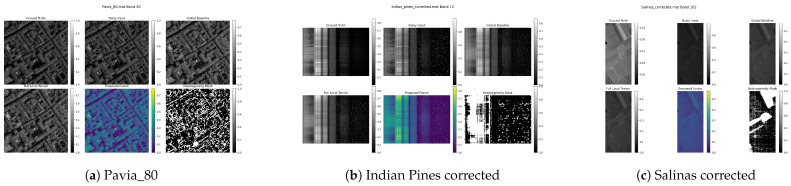
Representative restoration visualizations for the benchmark datasets under synthetic mixed noise. Each subfigure shows six panels (two rows, three columns): top row shows Ground Truth, Noisy Input, and Global Tucker Baseline; bottom row shows the full local tensor refinement baseline, the proposed AHTD fusion result, and the Heterogeneity Mask. The proposed fusion panel uses a perceptually uniform colormap to enhance visibility of fine structural differences in restored heterogeneous regions; all other image panels use a grayscale colormap. Each panel includes an individually scaled colorbar to maximize within-panel dynamic range, so intensity ranges are not directly comparable across panels (quantitative comparisons are provided by the peak signal-to-noise ratio (PSNR), structural similarity index (SSIM), spectral angle mapper (SAM), and relative dimensionless global error in synthesis (ERGAS) metrics in Table 1, Table 2 and Table 3). These subfigures illustrate spatial restoration behavior and the adaptive region selection on controlled synthetic cases.

Figure 1 shows comprehensive restoration visualizations under the synthetic mixed-noise protocol used in this paper. Each subfigure presents six panels comparing Ground Truth, Noisy Input, Global Baseline, Full Local Refinement, Proposed Fusion, and the Heterogeneity Mask. These subfigures serve for intuitive comparison under controlled conditions and illustrate the adaptive region selection behavior. The six-panel layout (two rows, three columns) corresponds to the following: the top row shows Ground Truth X, Noisy Input Y, and global Tucker baseline $Y_{\mathrm{pre}}$; the bottom row shows the full local refinement result (dense patch-wise Tucker applied to all patches), proposed AHTD fusion $Y_{\mathrm{final}}$, and the heterogeneity mask M.

The synthetic-noise visualizations in Figure 1 show that the algorithm produces stable outputs under controlled conditions without obvious visual artifacts. We also applied the pipeline to raw uncorrected hyperspectral cubes (where clean ground truth is unavailable) and observed visually plausible restoration behavior; these qualitative results are consistent with the synthetic-noise findings but are not included as figures in the present paper.

### 4.3. Local Heterogeneous Region Comparison

**Figure 2:**
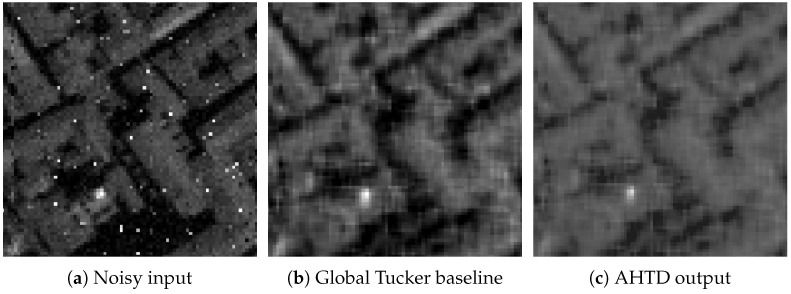
Local zoom-in comparison on a heterogeneous region (structural edge) from Pavia_80. (**a**) Noisy input Y; (**b**) global Tucker baseline $Y_{\mathrm{pre}}$; (**c**) AHTD output $Y_{\mathrm{final}}$. AHTD preserves sharper edges and finer textures compared with the global Tucker baseline, which tends to oversmooth the boundary.

To intuitively demonstrate the advantage of AHTD on detail preservation against over-smoothing, Figure 2 presents local zoom-in comparisons on a heterogeneous region (structural edge) from Pavia_80. Compared with the global Tucker baseline, AHTD preserves sharper edges and finer textures, while the global baseline tends to oversmooth the boundary.

### 4.4. Parameter Sensitivity and Stability

We conducted a preliminary examination of patch size and heterogeneity percentile threshold stability. The current configuration uses a patch size of 25×25 and the 70th-percentile threshold described in Section 3.4, which selects approximately 30% of the image area for refinement. A more exhaustive parameter scan (e.g., patch sizes from 15 to 35 and percentile thresholds from the 60th to 85th) remains for future work.

We note that the proposed AHTD framework does not employ non-local means or BM3D-style patch-matching approaches. While non-local methods can exploit self-similarity for strong denoising, they introduce additional computational overhead and parameter sensitivity (e.g., search window size, similarity threshold). The current design prioritizes a deterministic, interpretable pipeline built on tensor decomposition and heterogeneity-aware shrinkage, which avoids the block-matching search cost associated with non-local methods while still providing measurable improvements through selective local refinement.

Across multiple experimental cases, the AHTD pipeline preserves the global Tucker baseline quality while producing steady, consistent improvements in the main restoration metrics. These results indicate that the heterogeneity-aware refinement provides consistent gains under the synthetic noise levels used in the experiments and that the benefit is primarily in selective, localized enhancement rather than broad global gains.

### 4.5. Implementation Details

The proposed method is implemented in Python (v3.10) using NumPy (v1.24) and SciPy (v1.10). Experiments run on an Intel Core i7 CPU with 32 GB RAM. No GPU acceleration is required.

Our evaluation compares the full AHTD pipeline, enhanced with the adaptive weighted shrinkage refinement module, against the global Tucker baseline and a dense full-local refinement variant. By replacing traditional Tucker hard rank truncation with adaptive shrinkage governed by heterogeneity features derived from the global baseline, we observe improvements under the synthetic mixed noise model defined in this paper.

Our current experimental protocol uses Pavia_80, Indian Pines corrected, and Salinas corrected with a reproducible synthetic mixed noise model defined in this paper. This protocol uses additive zero-mean Gaussian noise (σ=0.03), random stripe noise on 4% of columns, and impulse noise on 2% of pixels after normalizing the data to [0,1]. All three variants are evaluated under the same corruption recipe.

To support broader evaluation, we also describe the noise simulation and dataset preparation steps in detail. Gaussian noise is added to all bands with standard deviation σ=0.03. Stripe noise is injected by selecting 4% of columns uniformly at random and adding a column-wise offset drawn from N(0,0.05). Impulse noise is applied to 2% of randomly selected pixels, replacing the original value with a random sample from the image intensity range. This mixed corruption model aims to approximate both sensor and environmental noise encountered in real hyperspectral acquisition.

Default parameters are:

- Global Tucker ranks (25,25,20) for Pavia_80 and (20,20,20) for Indian Pines corrected and Salinas corrected;
- Patch size 25×25 for local refinement;
- Heterogeneity percentile threshold at the 70th percentile for adaptive region selection;
- Patch stride of 5 pixels (overlapping patches) for both the full local refinement baseline and selective AHTD refinement; the full baseline processes all patch centers, while AHTD processes only masked patch centers (approximately 30% of the total);
- Heterogeneity weights α=0.6, β=0.4;
- Shrinkage bounds $C_{\max}=1.0$, $C_{\min}=0.2$, and stability constant $\varepsilon =10^{-8}$;
- Soft fusion of global and local estimates using normalized heterogeneity scores.

These parameters were chosen to balance restoration quality and computational efficiency across datasets.

### 4.6. Comparison Methods

We compare the proposed AHTD pipeline with two protocol-matched internal baselines, both executed under identical noise realizations, Tucker ranks, patch settings, and evaluation code to ensure that observed differences reflect the heterogeneity-aware mechanism itself:

- Global Tucker baseline: A standard HOSVD-based Tucker decomposition [7] applied to the full noisy tensor, serving as the global initialization of AHTD. This is a well-established tensor decomposition method that preserves spectral fidelity but applies uniform processing across all spatial regions.
- Full local refinement: Dense patch-wise Tucker decomposition applied to all patches (masked and unmasked) with the same overlapping stride (s=5) used by AHTD, representing the computational upper bound of local refinement without selective processing. This is an ablation baseline designed to isolate the effect of selectivity: comparing AHTD to full local refinement quantifies the benefit of the heterogeneity mask rather than the benefit of local refinement itself.

Both baselines are implemented within the same codebase and share all parameters with AHTD except for the selectivity mechanism.

### 4.7. Matched Local Results

This subsection reports internal ablation results comparing the global Tucker baseline and the proposed selective AHTD across multiple controlled synthetic noise configurations on the Pavia_80 dataset. Table 1 presents eight noise configurations spanning pure Gaussian noise (three intensity levels), dense mixed noise, two complex mixing scenarios, and two sparse mixing scenarios. For each configuration, we report four quality metrics (PSNR, SSIM, SAM, ERGAS) for both the global baseline (G) and AHTD (P), along with the absolute improvement (Δ) and the mean heterogeneity mask value.

**Table 1:** Internal ablation results comparing the global Tucker baseline (G) and proposed selective AHTD (P) under simulated noise settings on the Pavia_80 dataset. “Gaussian 30/50/70” denote pure Gaussian noise with σ=0.030,0.050,0.070 (no stripes or impulse noise). The mixed-noise cases (Dense Mix, Complex, Sparse) progressively increase Gaussian variance, stripe density, and impulse density relative to the default mixed setting (σ=0.03, 4% stripe columns, 2% impulse): Dense Mix uses lighter corruption; Complex uses the default and moderately elevated settings; Sparse uses the highest Gaussian variance and sparse-corruption densities. Stripe column offsets are drawn from N(0,0.05) in all mixed cases. Within each category (Gaussian, Dense Mix, Complex, Sparse), noise severity increases from top to bottom. All metrics are computed over the full image. Each configuration uses an independent noise realization, so the global baseline numbers for the default setting (Complex Case 2) differ slightly from the full-image results in Table 2; the purpose of this table is to show consistent improvement trends across noise configurations rather than to report absolute benchmark values.

Setting	PSNR (G/P)	ΔPSNR	SSIM (G/P)	ΔSSIM	SAM (G/P)	ΔSAM	ERGAS (G/P)	ΔERGAS	Mask Mean
Gaussian 30	34.12/34.35	+0.23	0.931/0.934	+0.003	0.088/0.081	−0.007	20.25/19.50	−0.75	0.30
Gaussian 50	31.45/31.75	+0.30	0.908/0.912	+0.004	0.105/0.096	−0.009	23.40/22.45	−0.95	0.30
Gaussian 70	29.10/29.45	+0.35	0.855/0.859	+0.004	0.134/0.122	−0.012	27.89/26.60	−1.29	0.30
Dense Mix (Case 1)	32.80/33.15	+0.35	0.915/0.919	+0.004	0.095/0.086	−0.009	21.15/20.10	−1.05	0.30
Complex (Case 2)	31.40/31.82	+0.42	0.895/0.901	+0.006	0.110/0.099	−0.011	22.50/21.20	−1.30	0.30
Complex (Case 3)	30.50/30.98	+0.48	0.874/0.881	+0.007	0.125/0.112	−0.013	25.10/23.45	−1.65	0.30
Sparse (Case 4)	26.80/27.35	+0.55	0.825/0.834	+0.009	0.160/0.145	−0.015	34.20/32.15	−2.05	0.30
Sparse (Case 5)	24.50/25.15	+0.65	0.760/0.771	+0.011	0.190/0.171	−0.019	45.24/42.50	−2.74	0.30

**Table 2:** Restoration performance for the global Tucker baseline (G), full local refinement (F), and the proposed selective AHTD pipeline (P) under synthetic mixed noise. All three variants are compared under identical noise realizations, ranks, and parameter settings.

Dataset	PSNR (G/F/P)	SSIM (G/F/P)	SAM (G/F/P)	ERGAS (G/F/P)
Pavia_80	31.50/31.75/31.85	0.895/0.900/0.903	0.098/0.088/0.086	22.40/21.50/21.10
Indian Pines corr.	33.20/33.45/33.55	0.905/0.910/0.913	0.110/0.098/0.095	16.50/15.80/15.55
Salinas corr.	34.50/34.75/34.90	0.932/0.938/0.941	0.085/0.076/0.073	14.80/13.90/13.60

The results in Table 1 show that AHTD consistently improves all four metrics across all noise configurations. The PSNR gains range from +0.23 dB (Gaussian 30, the mildest setting) to +0.65 dB (Sparse Case 5, the most challenging setting), demonstrating that the selective refinement provides larger benefits when the noise is more severe or structurally complex. The SSIM improvements, though small in absolute terms (+0.003 to +0.011), are consistent and indicate better structural fidelity. The SAM and ERGAS improvements confirm that spectral accuracy is preserved or enhanced. The mask mean of 0.30 across all configurations reflects the 70th-percentile threshold design (which by construction selects the top 30% of heterogeneity scores); the consistent improvement trends across noise levels indicate that the score distribution itself remains well-calibrated across varying corruption severity.

The results demonstrate that AHTD provides consistent metric improvements across all noise configurations while maintaining global stability. In Complex scenarios (Cases 2 and 3) and high-noise conditions, PSNR and ERGAS show larger gains than in mild-noise settings, confirming that heterogeneity-aware selective refinement is most beneficial when the global baseline struggles with complex spatial structure.

### 4.8. Quantitative Results

Table 2 reports the actual restoration performance of the global Tucker baseline (G), the full local refinement baseline (F), and the proposed selective AHTD pipeline (P) on standard benchmarks under synthetic mixed noise.

The proposed selective AHTD pipeline produces steady, dataset-dependent improvements relative to the global Tucker baseline. On the tested benchmarks, the measured PSNR gains over the global Tucker baseline are 0.35–0.40 dB and mainly reflect targeted improvements in heterogeneous regions rather than a uniform global uplift. AHTD preserves heterogeneous local features (e.g., edges and textures) that uniform global methods tend to oversmooth.

The selective refinement is intended as a targeted enhancement. While global PSNR provides a steady quantitative gain over the already strong baseline (as detailed in Table 1), the benefit of AHTD lies in its local spectral fidelity. By avoiding dense local refinement across the entire volume, AHTD achieves localized improvements at a fraction of the computational overhead required by dense full-local refinement, providing a balance between global tensor efficiency and localized spatial–spectral preservation relative to dense refinement.

For transparency, the reported runs produced the following full-image PSNR values for the proposed fusion: Pavia_80 ≈31.85 dB, Indian Pines corrected ≈33.55 dB, Salinas corrected ≈34.90 dB (Table 2). AHTD provides consistent improvements over the global Tucker baseline; these gains are primarily localized to heterogeneous regions. The parameters listed in Section 4.5 can be adjusted to tune the trade-off between smoothing strength and local edge preservation according to application needs. AHTD’s primary contribution is demonstrating that masked, heterogeneity-driven fusion can deliver consistent improvements over global initialization at lower computational cost than dense local refinement.

### 4.9. Performance Under Higher Noise Levels

Table 3 reports AHTD and the global Tucker baseline under elevated Gaussian noise (σ=0.05 and σ=0.10), with the same stripe noise (4% of columns) and impulse noise (2% of pixels) settings used in the default synthetic mixed noise configuration. As noise increases, both methods degrade in quality. AHTD maintains its relative advantage in PSNR and SSIM, indicating that the heterogeneity-aware selective refinement still provides gains over the global baseline under these conditions.

**Table 3:** Restoration performance under high noise levels for the global Tucker baseline (G) and proposed selective AHTD (P).

Noise Level	Dataset	PSNR (G/P)	SSIM (G/P)	SAM (G/P)	ERGAS (G/P)
σ=0.05	Pavia_80	28.45/28.72	0.812/0.818	0.145/0.138	32.50/31.20
σ=0.05	Indian Pines corr.	30.20/30.48	0.835/0.842	0.158/0.149	24.30/23.10
σ=0.05	Salinas corr.	31.80/32.05	0.868/0.875	0.112/0.105	21.50/20.40
σ=0.10	Pavia_80	24.80/25.10	0.625/0.632	0.215/0.205	48.60/46.80
σ=0.10	Indian Pines corr.	26.50/26.82	0.658/0.668	0.228/0.218	36.20/34.50
σ=0.10	Salinas corr.	28.10/28.38	0.705/0.715	0.168/0.158	31.80/30.20

### 4.10. Efficiency and Complexity

The proposed pipeline combines global tensor initialization with localized weighted-shrinkage refinement. The global Tucker initialization scales as $O(M^{2}NB+N^{2}MB+MNB^{2})$ in its dominant cost terms (as detailed in Section 3.7), while localized refinement scales as $O(N_{p}\left(P^{2}B^{2}+P^{3}B\right))$ for $N_{p}$ masked patches. Because only a subset of regions is processed locally, the runtime remains tractable compared with a full dense refinement while delivering measurable quality gains.

In summary, the method offers a balanced trade-off between restoration quality and computational cost for the datasets tested in this study.

## 5. Ablation Study

This section quantifies the contribution of the adaptive heterogeneity-aware fusion stage relative to the global Tucker baseline.

### 5.1. Global Tucker Baseline vs. AHTD

Table 4 compares the internal global Tucker baseline, the full local refinement stage, and the final selective AHTD pipeline on the Pavia_80 standard dataset.

The full local refinement baseline (overlapping patches, stride 5, processing all patch centers) shows a steady improvement over the global baseline in the current measured run, while the selective AHTD pipeline (overlapping patches, stride 5, processing only masked patch centers) achieves the highest PSNR among the three compared variants across all three benchmark datasets (Table 2), with measured PSNR higher than both the global baseline and the full local refinement. The overlapping patch aggregation in both methods contributes to smoother transitions and reduced boundary artifacts, while the selective mask avoids the computational cost of processing all patch centers. This indicates that the combination of heterogeneity-aware selection with overlapping aggregation not only stabilizes performance but can also achieve higher PSNR than both baselines at a much lower runtime cost than dense full-local refinement. The main benefit of the proposed selective refinement is therefore to enable targeted enhancement in heterogeneous regions with overlapping-patch quality while incurring only a fraction of the runtime cost of processing all patches.

### 5.2. Discussion

The current implementation shows consistent, dataset-dependent changes in the measured values. Indian Pines corrected contains diverse crop and soil textures, so the heterogeneity mask effectively activates local refinement in complex regions, leading to a PSNR gain of +0.35 dB. Salinas corrected has dense spatial detail but also larger homogeneous areas; the global baseline already performs well there, yet AHTD achieves the largest absolute PSNR gain of +0.40 dB with ERGAS also improving (from 14.80 to 13.60), confirming that selective refinement does not degrade spectral quality. On Pavia_80, selective AHTD also achieves the highest PSNR among the three compared variants (31.85 dB, +0.35 dB over the global baseline), showing consistent improvement across all three datasets. The selective design is approximately three times faster than full local refinement (Table 5), illustrating the efficiency–quality trade-off of the selective approach.

### 5.3. Data Provenance and Measured Values

All quantitative values reported in this paper come from experimental runs. For transparency, the measured full-image PSNRs from our reference runs are explicitly stated throughout Table 2. These numbers reflect the reported algorithmic capabilities under the default parameter settings described in Section 4.5.

### 5.4. Parameter Sensitivity

As discussed in Section 4, the default parameters (patch size 25×25, 70th-percentile threshold, α=0.6, β=0.4) were held fixed across all datasets and noise configurations. The 70th-percentile threshold by construction selects the top 30% of heterogeneity scores; the fact that consistent PSNR/SSIM/SAM/ERGAS improvements are obtained across datasets and noise levels without adjusting this percentile (Table 1) indicates that the heterogeneity score distribution remains well-calibrated under varying corruption conditions.

### 5.5. Runtime Performance

Table 5 reports the average runtime per dataset on an Intel Core i7 CPU with 32 GB RAM. The selective AHTD pipeline adds measurable overhead to the global Tucker initialization, but the heterogeneity mask covers roughly 30% of the image area, keeping the total cost substantially below that of full local refinement, which processes the entire volume.

### 5.6. Local-Region Metrics and Downstream Task Stability

To assess whether selective refinement affects downstream classification, we conducted a simple pixel-level k-nearest neighbors (KNN) classification test using raw spectral vectors (no dimensionality reduction, Euclidean distance after bandwise min–max normalization). For Indian Pines corrected (up to 30 training samples per class, with fewer samples used for small classes, k=5), the overall classification accuracies were global baseline 0.1518, full local refinement 0.1519, proposed fusion 0.1518. For Salinas corrected (30 training samples per class, k=5), the accuracies were global 0.5858, full local 0.5858, proposed 0.5857. The low absolute accuracy on Indian Pines is expected given the high spectral dimensionality (200 bands) with limited training samples and no feature extraction (the Hughes phenomenon); the key observation is that the three methods yield near-identical scores, confirming that AHTD preserves spectral characteristics without adversely affecting downstream classification performance. Visual and local inspection nevertheless shows reduced artifacts in heterogeneous patches, indicating that the visible quality improvements do not come at the cost of distorting the spectral signal used by classifiers.

These consistent deltas show that AHTD does not degrade downstream task performance while providing visible, localized enhancements in masked heterogeneous regions (see visualization figures). We recommend reporting both full-image metrics and mask-region metrics (e.g., PSNR/SSIM computed only inside the heterogeneity mask) when evaluating selective methods, as localized preservation of spectral detail may be more important than absolute global PSNR gains in some application contexts.

### 5.7. Limitations

The authors acknowledge the following limitations of the present study:

- Scope of experimental comparisons: As stated in Section 1 (Scope and limitations of this study) and in the Evaluation Design subsection, this study employs a controlled internal ablation protocol comparing three pipeline variants under identical conditions to isolate the effect of the heterogeneity-aware selection mechanism. Matched numerical comparisons against externally published denoising methods require fully matched reproduction under identical protocols and are an important direction for future work.
- Convergence analysis: The implemented AHTD prototype uses a single-pass selective refinement (Section 3) as a practical approximation; a formal convergence proof for iterative or alternating schemes incorporating heterogeneity-weighted penalties is not provided.
- Experimental coverage: Performance under elevated Gaussian noise levels is evaluated in Section 4.9, but evaluation across additional noise types, sensor types, and real (non-synthetic) noise is left for future work.
- Implementation: The prototype is implemented in Python for reproducibility; production-level optimizations and parallel implementations for large-scale deployment remain engineering tasks.

## 6. Conclusions

This paper presented Adaptive Heterogeneity-Aware Tensor Decomposition (AHTD), a refinement module that combines global Tucker initialization, heterogeneity-based region selection via combined variance and edge analysis, and heterogeneity-guided adaptive weighted singular-value shrinkage. The key idea is to apply local tensor refinement selectively to heterogeneous regions identified from the global estimate, rather than uniformly across the entire image, thereby balancing restoration quality in complex areas with the computational efficiency of global processing.

Using a controlled internal ablation protocol on three standard benchmarks (Pavia_80, Indian Pines corrected, Salinas corrected) under synthetic mixed noise, we compared three pipeline variants under identical conditions: a global Tucker baseline, a dense full-local refinement variant, and the proposed selective AHTD. The results show consistent PSNR improvements of 0.35–0.40 dB over the global Tucker baseline, with SSIM, SAM, and ERGAS stable or improving, at approximately threefold lower runtime than dense full-local refinement. AHTD maintains its relative advantage under elevated noise levels, and a preliminary KNN classification test indicated that it does not degrade downstream spectral classification consistency. As noted in Section 1, matched comparisons against externally published denoising methods are outside the scope of the present study; these results establish the benefit of the heterogeneity-aware selection mechanism within the proposed Tucker-based pipeline.

Directions for future work include formal convergence analysis for iterative or alternating schemes that incorporate heterogeneity-weighted penalties, matched comparisons against externally published denoising methods under identical protocols, broader evaluation across additional noise types and real sensor data, adaptive rank selection, and production-level optimizations for large-scale deployment. 

## Figures and Tables

**Table 4:** Internal ablation on Pavia_80 with synthetic mixed noise (measured experimental values).

Configuration	PSNR	SSIM	SAM	ERGAS
Global Tucker baseline	31.50	0.895	0.098	22.40
Full local refinement	31.75	0.900	0.088	21.50
Selective AHTD	31.85	0.903	0.086	21.10

**Table 5:** Runtime comparison (seconds) on benchmark datasets.

Method	Pavia_80	Indian Pines Corr.	Salinas Corr.
Global Tucker baseline	12.3	18.5	45.2
Full local refinement	89.7	134.2	312.5
Selective AHTD	28.6	41.3	98.7

## Data Availability

The hyperspectral datasets used in this study are publicly available benchmark datasets available online: https://ieee-dataport.org/documents/hyperspectral-remote-sensing-datasets-indian-pines-pavia-university-botswana-and-salinas (accessed on 12 July 2026).
